# Publisher Correction: Sensory attenuation in Parkinson’s disease is related to disease severity and dopamine dose

**DOI:** 10.1038/s41598-018-35439-8

**Published:** 2018-11-22

**Authors:** Noham Wolpe, Jiaxiang Zhang, Cristina Nombela, James N. Ingram, Daniel M. Wolpert, Cam-CAN, Lorraine K. Tyler, Carol Brayne, Edward T. Bullmore, Andrew C. Calder, Rhodri Cusack, Tim Dalgleish, John Duncan, Fiona E. Matthews, William D. Marslen-Wilson, Meredith A. Shafto, Teresa Cheung, Linda Geerligs, Anna McCarrey, Abdur Mustafa, Darren Price, David Samu, Matthias Treder, Kamen A. Tsvetanov, Janna van Belle, Nitin Williams, Lauren Bates, Andrew Gadie, Sofia Gerbase, Stanimira Georgieva, Claire Hanley, Beth Parkin, David Troy, Tibor Auer, Marta Correia, Lu Gao, Emma Green, Rafael Henriques, Jodie Allen, Gillian Amery, Liana Amunts, Anne Barcroft, Amanda Castle, Cheryl Dias, Jonathan Dowrick, Melissa Fair, Hayley Fisher, Anna Goulding, Adarsh Grewal, Geoff Hale, Andrew Hilton, Frances Johnson, Patricia Johnston, Thea Kavanagh-Williamson, Magdalena Kwasniewska, Alison McMinn, Kim Norman, Jessica Penrose, Fiona Roby, Diane Rowland, John Sargeant, Maggie Squire, Beth Stevens, Aldabra Stoddart, Cheryl Stone, Tracy Thompson, Ozlem Yazlik, Dan Barnes, Marie Dixon, Jaya Hillman, Joanne Mitchell, Laura Villis, James B. Rowe

**Affiliations:** 1 Department of Clinical Neurosciences, University of Cambridge, Cambridge, CB2 0SZ UK; 2 Medical Research Council Cognition and Brain Sciences Unit, Cambridge, CB2 7EF UK; 3 Cambridge Centre for Ageing and Neuroscience, University of Cambridge and MRC Cognition and Brain Sciences Unit, Cambridge, CB2 3EB UK; 4 Cardiff University Brain Research Imaging Centre, School of Psychology, Cardiff University, Cardiff, CF24 4HQ UK; 5 Computational and Biological Learning Laboratory, Department of Engineering, University of Cambridge, Cambridge, CB2 1PZ UK; 6 Zuckerman Mind Brain Behavior Institute, Department of Neuroscience, Columbia University, New York, United States

Correction to: *Scientific Reports* 10.1038/s41598-018-33678-3, published online 23 October 2018

The original version of this Article contained an error in the order of author names, which were incorrectly listed as ‘Noham Wolpe, Jiaxiang Zhang, Cristina Nombela, James N. Ingram, Daniel M. Wolpert, James B. Rowe & Cam-CAN’.

This error has now been corrected in the HTML version of this Article; the PDF version of the paper was correct from the time of publication.

